# Therapy and material choices in pulp exposure among public dentists in Norway

**DOI:** 10.2340/aos.v85.45574

**Published:** 2026-03-18

**Authors:** Bo Wold Nilsen, Nema Rashdan, Mudar Rashdan, Anca Virtej

**Affiliations:** Department of Clinical Dentistry, Faculty of Health Sciences, UiT The Arctic University of Norway, Tromsø, Norway

**Keywords:** Survey, vital pulp therapy, biomaterial, public dental service

## Abstract

**Objective:**

This study aimed to investigate the following: (1) the preferences of public dentists for procedures and materials in carious or non-carious pulp exposure of permanent teeth; (2) how factors such as clinical experience, scientific literature reading, and material availability affect their choices; (3) the self-assessed risk of root canal treatment after pulp capping.

**Materials and methods:**

An online questionnaire consisting of 20 multiple choice and open-ended questions was e-mailed to Norwegian public dental clinics. It assessed dentists’ material preferences for direct pulp capping for carious or non-carious exposures, alongside factors such as years since graduation, scientific literature engagement, availability of materials, and clinical scenario choices. Respondents were also asked to estimate and reason long-term outcomes of their chosen materials. Standardized case descriptions ensured shared clinical understanding. Data were statistically analyzed and *p*-values ≤ 0.05 were considered statistically significant.

**Results:**

A total of 218 (23.9%) dentists responded. Direct pulp capping was preferred by 65% of respondents, with chemically curing materials – primarily calcium hydroxide – being most used. Chi-square analyses showed that dentists with fewer years of experience and those who had read scientific literature were more likely to prefer calcium silicate materials; however, these variables were not statistically significant predictors in the logistic regression models. Material availability was the strongest predictor of preference, with significantly increased odds of selecting calcium silicates or light-curing materials when available. ‘Satisfied with clinical results for the chosen material’ was the most frequently reported reason for material selection.

**Conclusion:**

Most respondents in this study preferred calcium hydroxide for direct pulp capping in permanent teeth with closed apices, despite the superior clinical outcomes of hydraulic calcium silicates. Material availability in the clinic was the primary factor influencing dentists’ choices, surpassing clinical experience and scientific literature engagement.

## Introduction

The dental pulp is a richly vascularized and innervated connective tissue that is crucial for tooth development, nutrition, and sensitivity. It is surrounded by dental hard tissues, which comprise a rigid physical barrier against pathogenic challenges and injuries. Both innate and adaptive immune mechanisms are prevalent in the dental pulp, and their interplay with the dental pulp-specialized cells, the odontoblasts, aims at defending the pulp from external irritants. Once the integrity of hard tissue barrier is breached through carious, mechanical or traumatic processes, noxious elements such as microorganisms will gain entry to the tissue [1, 2]. This leads to various degrees of pulp inflammation, and eventually to microbial pulp exposure.

The prevalence of deep caries (untreated and/or treated) remains high and varies depending on geography and age group, with epidemiological data indicating a 20% prevalence among adolescents in European Nordic countries [3, 4].

When pulp exposure occurs, it can be managed with vital pulp therapy such as direct pulp capping or pulpotomy which aims at maintaining pulp tissue viability, or with pulpectomy. While some studies advocate vital pulp therapy regardless of the inflammatory state of the pulp [5–7], the European Society of Endodontology still recommends that the tooth must not show symptoms of irreversible pulpitis [8]. This recommendation, which Norwegian dental practitioners also adhere to, recognizes the lack of high-quality clinical trials comparing different types of interventions [9]. Direct pulp capping is a treatment method where the exposed pulp is covered with a biomaterial that stimulates healing and the formation of reparative dentin [10]. Pulpotomy relies on the same principle; however, the procedure implies removal of inflamed pulp tissue prior to the application of various biomaterials inducive of hard tissue barriers. The prognosis for these treatments depends on several factors such as pulp status, bleeding control, the patient’s age, tooth’s maturity, aseptic conditions [11], use of magnification, and also the biomaterial itself [12, 13].

There are different biomaterials for direct pulp capping or pulpotomy, including chemically curing materials such as calcium hydroxide and hydraulic calcium silicate cements like mineral trioxide aggregate (MTA), as well as light-curing materials based on the same chemicals found in chemically curing materials [14]. Calcium hydroxide has been used since 1921 and promotes an inflammatory response that leads to dentin formation, but it has disadvantages such as water solubility and reduced sealing ability. MTA, introduced in the 1990s, has shown to generate better sealing and induce thicker dentin bridges when used. However, it is more expensive, harder to use, and can discolor teeth [15]. Tricalcium silicate cements are another group of materials (e.g. Biodentine) similar to MTA, but with a shorter setting time and have less potential for tooth discoloration [14, 16]. Light-curing resin-modified calcium silicate materials, like Theracal LC, are easier to handle and do not require manual mixing, but there has been discussion in the literature about whether their physical and chemical properties make them suitable for direct pulp capping [17, 18]. With regard to clinical outcomes, MTA and tricalcium silicates have, in general, shown better results than calcium hydroxide [15]. Fewer studies have focused on light-curing materials, with contradictory results. As a direct pulp capping material, Theracal was deemed both more [19] and less successful [20] in achieving a continuous dentin bridge formation. A recently published systematic review concluded that Theracal LC had clinical efficacy comparable to chemically curing materials [21]; however, another study showed that these materials are less suitable for direct pulp capping than conventional biomaterials in the long term [22].

Despite research showing superior outcomes of hydraulic calcium silicate cements in vital pulp therapy, an investigation published in 2017 indicated that direct pulp capping with the application of calcium hydroxide is the preferred method for treating carious pulp exposure among dentists in Norway, Germany, and France [23]. Similar findings were reported by a Finnish study [24]. However, a recent study has identified that number of years in dental practice and ‘recommended scientific publications’ influence management preferences of deep caries lesions among Lithuanian dentists [25].

While hydraulic calcium silicates are strongly recommended, guidelines on vital pulp therapy are still permissive with regards to types of materials that can be employed [8]. Several factors may influence the choice of biomaterials in a clinical setting. Most studies have not analyzed if a non-carious exposure may influence material choice. The impact of material availability in public dental clinics and the perceived clinical effectiveness on material selection remain unclear. The factors influencing the choice between chemically curing and light-curing materials have neither been investigated. We hypothesized that the type of pulp exposure (carious vs. non-carious), the dentist’s experience, reading of scientific literature, and the availability of the pulp capping material will influence the material choice for vital pulp therapy in asymptomatic teeth with closed apex in Norwegian public dental clinics.

### Aims

This study aimed to (1) explore Norwegian public dentists’ preferences for materials and procedures in non-carious and carious pulp exposure in asymptomatic permanent teeth, (2) examine the relationship between the dentists’ clinical experience, reading of scientific literature, the availability of materials at the clinic, and their choice of materials, and (3) map the self-assessed risk of root canal treatment after performing pulp capping with the preferred material.

## Materials and methods

An online questionnaire was developed using Nettskjema, a web-based survey tool developed by the University of Oslo. The questionnaire was tested among clinical instructors at the Department of Clinical Dentistry, UiT the Arctic University of Norway and the content was modified according to the received feedback. The design of the survey was based on the layout of previous surveys on the same topic from 2013 to 2017 [23, 26], but the options related to light-curing capping materials were expanded. In addition, non-carious exposures were mapped individually from carious pulp exposures. The questionnaire consisted of 1) a section explaining the purpose of the questionnaire and emphasizing anonymity; 2) a section to investigate the demographic data of participants which included years since graduation and reading of scientific articles on dental materials used in direct pulp capping; 3) a section to examine treatment and material preferences managing pulp exposures, as well as perceived treatment success with the preferred method, and the reason for preferring the material. (Supplemental data). The questionnaire had no option of review steps before submission. Since no personal data of participants was handled, an application to the Data Protection Official for Research was not required.

Contact information of all public dental clinics was acquired via the website of the Norwegian Dental Association. An invitation to the survey was sent via email to the clinics. The email informed about the goals of the survey, type of data collection and included an attached document with a QR code to the questionnaire. Additionally, a document (with a QR code) was provided to the clinics for increased accessibility to the dentists working at the clinics. All dental clinics were also contacted by phone to enquire whether they would participate in the survey, as well as to provide the number of employed dental practitioners (which were used to calculate response rate). As such, the response rate was based on the total number of dentists employed in each clinic. The data was collected between August 2023 and October 2023. The data collected did not include any personal information of the respondents, as informed in the questionnaire. No incentives were offered to the respondents.

The collected data was organized using a spreadsheet (Microsoft Excel, Version 2412 for Microsoft 365, Microsoft, Redwood, USA) and analyzed using SPSS (Version 26, IBM, Armonk, NY, USA). Descriptive statistics were generated, and chi-square tests were used to examine the relationship between the dentists’ clinical experience (range of years in practice), reading of scientific literature, material availability in the clinic, and choice of materials. The perceived success rate of treatment in carious versus non-carious exposures was assessed using a Mann-Whitney U test, as the assumption of normality required for a t-test was not met. Normality and Levene’s tests were initially performed to evaluate the appropriateness of using a t-test. Logistic regression analyses were performed to evaluate the predictors of preference for calcium silicate materials in both carious and non-carious pulp exposures, with years since graduation, engagement with scientific literature, and material availability as explanatory variables. Preference for light-curing materials was done in a similar manner. *P*-values ≤ 0.05 were considered statistically significant.

## Results

The survey was distributed to 898 dentists in the public dental health service, of which 218 responded. Three participants were excluded because they did not complete the survey. The final response rate was calculated to be 23.9%. Table 1 provides an overview of respondents’ clinical experience and their use of scientific literature.

**Table 1:** Overview of respondents’ clinical experience and the use of scientific literature.

		Have you read scientific articles on dental materials used for direct pulp capping in the last 5 years?		
		Yes	No	Total
How many years has it been since you graduated as a dentist?	< 5 years	51 (86.4%)	8 (13.6%)	59
	5–10 years	35 (70%)	15 (30%)	50
	10–20 years	39 (65%)	21 (35%)	60
	More than 20 years	35 (76.1%)	11 (23.9%)	46
**Total**		160 (74.4%)	55 (25.6%)	215

Number (*N*) with percentage in brackets.

**Figure 1:**
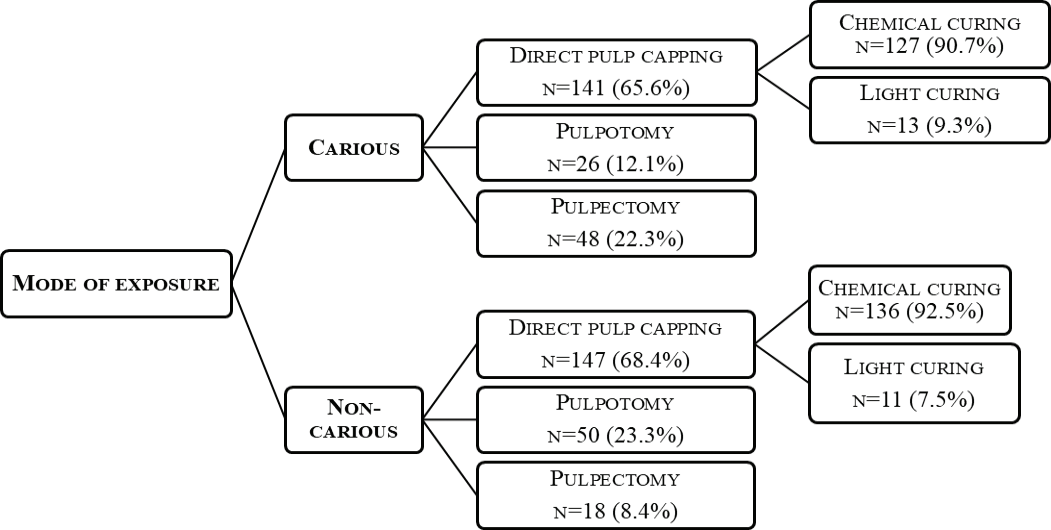
Overview of preferences for vital pulp therapy and material selection in direct pulp capping for carious and non‑carious exposures

In the case of carious pulp exposure of an asymptomatic permanent tooth with completed root development, it was found that 65.6%, 12.1%, and 22.3% of respondents would perform direct pulp capping, pulpotomy, and pulpectomy, respectively. For non-carious exposure, 68.4%, 23.3%, and 8.4% would perform direct pulp capping, pulpotomy, and pulpectomy, respectively. Of the participants who would perform direct pulp capping for carious and non-carious exposures, around 90 % preferred chemically curing materials (Figure 1).

The material that was most available at public dental clinics in Norway was calcium hydroxide (~95%) (Table 2). About 30% of the clinics had a light-curing material option. For carious and non-carious exposures, respectively, 48.2% and 49.7% preferred calcium hydroxide cements for direct pulp capping, while 39% and 37.5% would have preferred calcium silicate-based cements. A statistically significant relationship was found between material preference and availability at the clinic (*p* < 0.001).

**Table 2:** Distribution of the participants’ preference of materials (first choice) when handling exposed pulp (carious and non-carious).

Material	Availability in the clinic	Carious exposure (material preference)	Non-carious exposure (material preference)
Calcium hydroxide, e.g. Dycal , Life	203 (94.4%)	68 (48.2%)	73 (49.7%)
MTA, e.g. ProRoot MTA	55 (25.6%)	10 (7.1%)	7 (4.8%)
Other calcium silicate-based cements, e.g. MTA-Angelus, Biodentine	103 (47.9%)	45 (31.9%)	48 (32.7%)
Resin modified calcium hydroxide, e.g. Prisma VLC Dycal, Ultra-Blend Plus, Calcimol LC	38 (17.7%)	7 (5.0%)	5 (3.4%)
Resin modified calcium silicate, e.g. Theracal LC	34 (15.8%)	6 (4.3%)	6 (4.1%)
Cavity lining, e.g. IRM or GIC	-	3 (1.4%)	4 (1.9%)
None, bonds directly to the dentin	-	1 (0.5%)	-
Other	-	1 (0.7%)	4 (2.7%)

MTA: mineral trioxide aggregate; IRM: Intermediate Restorative Material ; GIC: Glass Ionomer Cement .

Number (*N*) with percentage in brackets.

Less experienced dentists had read significantly more literature on the topic compared to those with over 5 years of experience (*p* = 0.013). When examining preferences for chemically curing versus light-curing materials, no statistically significant difference was observed based on years of practice or the amount of literature read (Table 3). However, a statistically significant correlation was found between engagement with literature and the preference for calcium silicates as a direct capping material for carious exposures (*p* = 0.017) and non-carious exposures (*p* = 0.031) (Table 4).

**Table 3:** Cross table: material preference for direct pulp capping in carious and non-carious exposures with the number of years since dentists completed their training and professional update.

Carious exposures					
		Preference for chemically curing materials	Preference for light curing materials	Total	*P*-value
How many years has it been since you graduated as a dentist?	5 years or less experience	26 (96.3%)	1 (3.7%)	27	0.26
	More than 5 years experience	101 (89.4%)	12 (10.6%)	113	
Have you read scientific articles on dental materials used for direct pulp capping in the last 5 years?	No	29 (87.9%)	4 (12.1%)	33	0.52
	Yes	98 (91.6%)	9 (8.4%)	107	
Are light curing capping materials available at the clinic?	Yes No	38 (76%) 89 (98.9%)	12 (24%) 1 (1.1%)	50 90	< 0.001
Non- carious exposures					
How many years has it been since you graduated as a dentist?	5 years or less experience	30 (100%)	0	30	0.08
	More than 5 years experience	106 (90.6%)	11 (9.4%)	117	
Have you read scientific articles on dental materials used for direct pulp capping in the last 5 years?	No	40 (95.2%)	2 (4.8%)	42	0.42
	Yes	96 (91.4%)	9 (8.6%)	105	
Are light curing capping materials available at the clinic?	Yes No	39 (81.3%) 97 (98%)	9 (18%) 2 (2%)	48 99	< 0.001

Number (*N*) with percentage in brackets.

**Table 4:** Cross table: material preference and availability at the clinic grouped after calcium silicate/non calcium silicate for direct pulp capping in carious and non-carious exposures with the number of years since dentists completed their training and professional update.

Carious exposures					
		Preference for non-calcium silicate material	Preference for calcium silicate materials	Total	*P*-value
How many years has it been since you graduated as a dentist?	5 years or less experience More than 5 years experience	71 (62.8%) 15 (55.6%)	43 (38.1%) 12 (44.4%)	113 27	0.519
Have you read scientific articles on dental materials used for direct pulp capping in the last 5 years?	No Yes	26 (78.8%) 60 (56.1%)	7 (21.2%) 48 (43.9%)	33 107	0.017
Are calcium silicates available at the clinic?	Yes No	49 (98.0%) 37 (41.1%)	2 (2.0%) 53 (58.9%)	50 90	< 0.001
Non- carious exposures					
How many years has it been since you graduated as a dentist?	5 years or less experience More than 5 years experience	75 (64.1%) 17 (56.7%)	42 (35.9%) 13 (43.3%)	117 30	0.453
Have you read scientific articles on dental materials used for direct pulp capping in the last 5 years?	No Yes	32 (76.2%) 60 (57.1%)	10 (23.8%) 45 (42.9%)	42 105	0.031
Are calcium silicates available at the clinic?	Yes No	55 (96.5%) 37 (41.1%)	2 (3.5%) 53 (58.9%)	57 90	< 0.001

Number (*N*) with percentage in brackets.

Logistic regression analyses for both non-carious and carious pulp exposures identified the availability of calcium silicate and light-curing materials as the strongest and most significant predictors of preference. Other factors, such as years since graduation and engagement with scientific literature, were not significant in any of the models. Full details are presented in Table 5.

**Table 5:** Multivariable logistic regression results for predictors of material preference in non-carious and carious pulp exposures.

Exposure type	Material	Predictor	OR	95% CI	*p*-value	Nagelkerke *R*²
Non-carious	Calcium silicate	Years since graduation	3.071	0.906–10.412	0.072	0.455
		Engagement with literature	1.422	0.533–3.796	0.483	
		Availability	48.930	10.245–233.699	< 0.001	
	Light-curing	Years since graduation	0.000	0.000	0.998	0.271
		Engagement with literature	2.146	0.413–11.148	0.364	
		Availability	10.435	2.123–51.277	0.004	
Carious	Calcium silicate	Years since graduation	1.773	0.584–5.380	0.312	0.415
		Engagement with literature	1.415	0.462–4.333	0.544	
		Availability	34.713	7.709–156.315	< 0.001	
	Light-curing	Years since graduation	0.332	0.038–2.927	0.321	0.320
		Engagement with literature	0.608	0.150–2.465	0.486	
		Availability	29.230	3.634–235.114	0.002	

Table 6 provides an overview of the self-assessed risk of root canal treatment after carious and non-carious direct pulp capping procedures with different materials. When materials were categorized into light-curing and chemically curing, a Mann-Whitney U test revealed no statistically significant difference in the self-assessed risk of long-term root canal treatment for carious exposure. The mean risk for light-curing materials was 25.7 (standard deviation [SD] = 19.4), while for chemically curing materials, it was 40.1 (SD = 22.4), with a *p*-value of 0.224. Similarly, for non-carious exposures, the mean risk for light-curing materials was 29.5 (SD = 19.5) compared to 38.6 (SD = 23.2) for chemically curing materials, with a *p*-value of 0.527, indicating no statistically significant difference. Additionally, there were no statistically significant differences when comparing calcium silicates versus all materials, and calcium silicates versus calcium hydroxide (for both non-carious and carious exposure).

**Table 6:** The table shows the average self-assessed risk of root filling in the long term after direct pulp capping with the specified preferred material (Mean ± standard deviation expressed as percentages).

Material	Carious exposure	Non-carious exposure
1. Calcium hydroxide, e.g. Dycal , Life	40.5 ± 22.2	35.3 ± 22.2
2. MTA, e.g. ProRoot MTA	37.5 ± 17.9	20.3 ± 12.9
3. Other calcium silicate-based cements, e.g. MTA-Angelus, Biodentine	40.2 ± 24.3	41.3 ± 27.4
4. Resin modified calcium hydroxide, e.g. Prisma VLC Dycal , Ultra-Blend Plus, Calcimol LC	21.5 ± 15.6	27.8 ± 17.7
5. Resin modified calcium silicate, e.g. Theracal LC	30.0 ± 23.4	30.0 ± 19.6
6. Cavity lining, e.g. IRM or GIC	-	50.5 ± 28.9
Calcium silicates (2 and 3)	39.6 ± 23	37.9 ± 26
All materials except Calcium silicates (1,4,5,6)	38.4 ± 22	37.7 ± 21

MTA: mineral trioxide aggregate; IRM: Intermediate Restorative Material; GIC: Glass Ionomer Cement.

Reasons for preferences of materials are presented in Tables 7 and 8. Many seemed to prefer chemically curing options over light-curing materials, mostly due to their satisfaction with the clinical performance of the chemically curing material. Relatively few reported that factors such as lack of clinical studies of light-curing alternatives, or concerns regarding biocompatibility, were among reasons for preferring chemically curing materials. 24–40% reported that reduced availability of light-curing alternatives was a reason for their preference of chemically curing materials. For those who had light-curing materials as their first choice, the two most reported reasons were ‘satisfied with clinical results of light-curing capping materials’ and ‘light-curing capping materials bond better to permanent filling materials’.

**Table 7:** Reasons reported by participants for preferring chemical-curing capping materials over light-curing materials.

	Carious exposure	Non-carious exposure
Calcium hydroxide, e.g. Dycal , Life	68 (-)	73 (-)
Satisfied with clinical results for chemically curing capping materials	43 (63%)	46 (63%)
Poor clinical experience with light-curing capping materials	8 (12%)	7 (10%)
Few clinical studies on light-curing capping materials	8 (12%)	10 (14%)
Light-curing capping materials are not available at the clinic	17 (25%)	22 (30%)
Resin leads to pulp toxicity	11 (16%)	9 (12%)
Induction of heat during light curing	11 (16%)	10 (14%)
Other	16 (24%)	7 (9.6%)
MTA, e.g. ProRoot MTA	10 (-)	7 (-)
Satisfied with clinical results for chemically curing capping materials	7 (70%)	5 (71%)
Poor clinical experience with light-curing capping materials	0 (0%)	1 (14%)
Few clinical studies on light-curing capping materials	3 (30%)	0 (0%)
Light-curing capping materials are not available at the clinic	4 (40%)	2 (29%)
Resin leads to pulp toxicity	1 (10%)	2 (29%)
Induction of heat during light curing	3 (30%)	4 (57%)
Other	0 (0%)	0 (0%)
Orther calcium silicate-based cements, e.g. MTA-Angelus, Biodentine	45 (-)	48 (-)
Satisfied with clinical results for chemically curing capping materials	28 (62%)	33 (67%)
Poor clinical experience with light-curing capping materials	5 (11%)	7 (15%)
Few clinical studies on light-curing capping materials	5 (11%)	5 (10%)
Light-curing capping materials are not available at the clinic	11 (24%)	10 (21%)
Resin leads to pulp toxicity	22 (49%)	18 (38%)
Induction of heat during light curing	21 (47%)	14 (29%)
Other	3 (7%)	5 (10%)

MTA: mineral trioxide aggregate.

Multiple answers per participant possible. Number (*N*) with percentage of those who prefer the material (and state this reason) in brackets.

**Table 8:** Reasons reported by the participants for preferring light-curing capping materials over chemically curing materials.

	Carious exposure	Non-carious exposure
Resin modified calcium hydroxide, e.g. Prisma VLC Dycal, Ultra-Blend Plus, Calcimol LC	7 (-)	5 (-)
Satisfied with clinical results for light-curing capping materials	5 (71%)	5 (100%)
The chemically curing covering materials are difficult to use	1 (14%)	1 (20%)
Clinical studies show better or equally good results for light-curing covering materials	2 (29%)	1 (20%)
Bad clinical experience with the chemically hardening capping materials	0 (0%)	0 (0%)
The light-curing capping materials bond better to permanent filling materials	3 (43%)	3 (60%)
Other	2 (29%)	0 (0%)
Resin modified calcium silicate, e.g. Theracal LC	6 (-)	6 (-)
Satisfied with clinical results for light-curing capping materials	4 (67%)	5 (83%)
The chemically curing covering materials are difficult to use	3 (50%)	2 (33%)
Clinical studies show better or equally good results for light-curing covering materials	1 (17%)	0 (0%)
Bad clinical experience with the chemically hardening capping materials	0 (0%)	0 (0%)
The light-curing capping materials bond better to permanent filling materials	2 (33%)	1 (16.7%)

Multiple answers per participant possible. Number (*N*) with percentage of those who prefer the material (and state this reason) in brackets.

## Discussion

This study aimed to investigate Norwegian public dentists’ preferences and decision-making in the management of pulp exposures in asymptomatic permanent teeth. Specifically, it explored: (1) their choice of materials and procedures for carious and non-carious pulp exposures, (2) how clinical experience, engagement with scientific literature, and material availability influence these choices, and (3) their self-assessed risk of root canal treatment following pulp capping.

Direct pulp capping was preferred by the majority of respondents for both carious and non-carious exposures, with chemically curing materials – primarily calcium hydroxide – most commonly used. Despite calcium silicates being considered the ‘gold standard’, fewer than 40% of respondents selected them. ‘Satisfied with clinical results for the chosen material’ was the most frequently reported reason for material selection.

Availability of materials at the clinic emerged as the strongest and most consistent predictor of dentists’ preferences, surpassing both clinical experience and engagement with scientific literature. The odds of preferring calcium silicate materials were 49-fold greater for non-carious exposures and 35-fold greater for carious exposures when these materials were available. Similarly, availability strongly influenced preferences for light-curing materials. Chi-square analyses showed significant associations between engagement with scientific literature and preference for calcium silicates; however, these associations were not statistically significant in the logistic regression models, indicating that availability may be the dominant factor when multiple variables are considered.

Regarding self-assessed risk of root canal treatment following direct pulp capping, light-curing materials were associated with slightly lower estimated risk compared to chemically curing materials. However, no statistically significant differences were found between material types for either carious or non-carious exposures.

Our findings have certain limitations. The response rate in this study was 23.9%, which is slightly lower than the response rate in some similar studies. For example, in a survey from Finland on this topic, the response rate was 32% [24], while a multinational survey included 34% of Norwegian respondents [23]. Those who completed the survey may have had a particular interest in the topic or feel confident enough to respond to it. Therefore, one should be cautious regarding the generalization of the study’s results to all public dentists in Norway. A further limitation of this study is that it was not investigated in which county or region the participants work. Thus, we cannot determine whether the relatively few clinicians that preferred light-curing capping materials came from a certain clinic or region. Furthermore, teeth with completed root development could have been more clearly defined, as its definition may have been interpreted differently by the clinicians. Previous studies and guidelines point to better prognosis of direct pulp capping in patients younger than 40 years of age [14]. In a questionnaire study, the age of the patient was amongst the most reported patient-related factors influencing dentists’ treatment decisions for deep carious lesions [25]. However, several studies state that the outcome of vital pulp therapy is not influenced by age and may be considered as a successful treatment option in patients of all ages [27–29]. Thus, relevant patient factors, such as age, systemic health, the tooth’s response to sensibility tests as well as the radiographic periapical status may have been stated, to provide a broader understanding of factors influencing material preference among respondents in our study. Furthermore, the European Society of Endodontology emphasizes that rubber dam use and aseptic techniques are highly important for the success of vital pulp therapy procedures [8]. The placement of a rubber dam during endodontic treatments can vary [30, 31]. In our study, it is unknown how many dentists performed vital pulp treatments under strict aseptic conditions, which may have affected the perceived success rate of the direct capping procedures.

Pulp exposure may be treated with vital pulp therapies or root canal treatments. In this study, 65.6% of participants chose direct pulp capping for carious exposures and 68.4% for non-carious exposures, aligning closely with a multinational study where 68.4% of Norwegian dentists chose this treatment for a carious exposure scenario [23]. However, only 51% in Northern Norway selected this option under the same conditions, indicating potential regional differences or a change over time [26]. We lack studies on non-carious exposures, but in our study the difference was minimal in the proportion of dentists performing pulp capping. Direct pulp capping can be considered a viable treatment option of small pulp exposures treated shortly after a traumatic incident [32]. Pulp preservation by partial pulpotomy is a valuable treatment option in the majority of non-carious pulp exposures, supported by studies which indicated a higher rate of pulp necrosis following direct pulp capping compared to partial or complete pulpotomy [33, 34]. Previous studies have pointed out variations among dental practitioners’ knowledge on the importance of pulp preservation, with high variability on opting for pulpotomy as an adequate permanent treatment in cases of traumatic pulp exposures [35, 36]. The alternative to perform pulpectomy in these types of exposures was picked by less than 10% of dentists included in the current survey, indicating diverse opinions on the approach to non-carious pulp exposures.

Concerning materials for pulp capping, a meta-analysis [15] showed that direct pulp capping with MTA and tricalcium silicates have a higher success rate compared to calcium hydroxide. Despite this, MTA was among the least chosen chemically curing materials with 7.1% and 4.8% for carious and non-carious exposures, respectively; nevertheless, other calcium silicate materials were preferred by over 30% of participants in our study. The largest proportion of participants preferred the use of calcium hydroxide for direct pulp capping with 48.2% and 49.7% for carious and non-carious exposures, respectively. These results are in line with results from the Norwegian part of a larger multinational study, where most participants preferred calcium hydroxide-based cements for direct pulp capping in adults [23]. Contrary, Spanish dentists seemed to prefer the use of hydraulic calcium silicate cements, specifically Biodentine, in vital pulp therapy of permanent teeth following carious exposures [37]. Only 9.3% and 7.5% of participants in our study would choose light-curing materials for direct pulp capping for carious and non-carious exposures, respectively (Table 2).

Our study investigated the correlation between dentists’ work experience and their engagement with professional literature. We found that less experienced dentists read more scientific literature. This observation is in line with another Norwegian study, that more recently graduated dentists are more likely to stay informed with modern endodontic treatment routines compared to their more experienced colleagues [30]. In our study, reviewing literature had an impact on the preference for calcium silicates for carious as well as non-carious exposures. It was observed that most participants preferred chemically curing over light-curing materials for handling exposed pulp in asymptomatic permanent teeth with closed apex. The reason for so few practitioners using light-curing materials may be associated with the less successful clinical outcomes of these materials [22, 38], the biological concerns raised for these materials [17, 39], or rather their availability for the dentists.

Calcium hydroxide-based chemically curing materials were most available to participants, while they were also the most used materials for direct pulp capping. About 3 in 4 had a hydraulic calcium silicate available. About 30% had a light-curing capping material available. It is worth noting that light-curing capping materials are not a homogenous group, and they have other indications than direct pulp capping. For example, the resin modified calcium hydroxide Ultra-Blend plus is only indicated for limited non-carious pulp exposures, while Calcimol – a material with similar composition [17] – is only indicated for indirect pulp capping. On the other hand, Theracal LC, perhaps the most studied light-curing capping material, is indicated for all types of direct capping procedures. A possible explanation for the most preferred material being chemically curing calcium hydroxide may also be of a financial nature. A systematic review and meta-analysis concluded that pulp capping with calcium hydroxide is preferred over MTA in dental practice, and that factors like costs could be held accountable [40]. Costs may thus influence the availability of materials to the dental practitioners.

Regarding the risk of root canal treatment after using the materials, the results showed that for carious exposures, dentists who responded to the survey assessed a non-significant, higher risk of root canal treatment when using chemically curing materials compared to light-curing materials. A reason for this finding could be related to very few respondents using light-curing materials.

In a study conducted on humans where Single Bond Universal was used directly on exposed pulp, light microscopy showed subclinical bonding errors, despite the absence of clinical symptoms up to 30 days after treatment [41]. A study comparing Theracal LC with calcium silicates showed that even though teeth were asymptomatic 8 weeks after the treatment, the pulp organization and dentinal bridge formation had an inferior outcome in teeth that had been treated with Theracal LC [38]. Similarly, a randomized clinical study showed that the short-term success rate of Theracal LC is on the same level as that for Biodentine and MTA, but in the long term, it is somewhat reduced compared to the other materials [19]. Unsuccessful vital pulp therapy in mature teeth results in pulp necrosis, chronic periapical inflammation with possible systemic implications, bone loss, and the need for further endodontic and/or surgical procedures. In case of teeth in need of apexification, the choice of the correct material may be important to preserve pulp vitality, ensure root development and long-term tooth survival. Therefore, the ongoing follow-up of patients over time is of utmost importance, as it can influence dentists’ perceptions of the success and failure rates of the material and procedure.

## Conclusion

Norwegian public dentists most frequently preferred calcium hydroxide for direct pulp capping, despite calcium silicates being associated with better clinical outcomes. The findings of this study suggest that material availability is the strongest predictor of preference, outweighing the influence of both clinical experience and engagement with scientific literature. Based on these results, improving access to recommended materials, such as calcium silicates, may support the adoption of evidence-based practices.

## Supplementary Material


