# Supplementary material for: Therapy and material choices in pulp exposure among public dentists in Norway
Source: Acta Odontol Scand. 2026 Mar 18;85:44574. doi: 10.2340/aos.v85.45574 (PMC13005172; doi:10.2340/aos.v85.45574)
Supplement: Supplementary file 1 [file AOS-85-45574-s1.pdf]

Machine Translated by Google

Nettskjema

Supplementary material has been published as submitted. It has not been copyedited or typeset by Acta Odontologica Scandinavica.

Preferences for materials used for direct pulp capping in case of carious and mechanical exposures

The aim of this survey is to investigate dentists' choice of materials for pulp exposure.
The survey takes approximately 3 minutes to complete and does not collect any personally sensitive information.

How many years has it been since you graduated as a dentist?

<5 years

5 - 10 years

10 - 20 years

> 20 years

Have you read scientific articles about dental materials used for direct pulp capping in the last 5 years?

Yes

No

What capping materials do you have available at the clinic?

You can choose several options

Calcium hydroxide, e.g.: Dycal, Life

Resin modified calcium hydroxide, e.g.: Prisma VLC Dycal, Ultra-Blend Plus, Calcimol LC MTA, e.g.:

ProRoot MTA Calcium

silicate-based cements, e.g.: MTA-Angelus, Biodentin Resin modified

calcium silicate, e.g.: Theracal LC

Other

What other material?

This item is only displayed if the option "Other" is selected in the question "What capping materials do you have available at the clinic?"

If you expose the pulp during caries excavation in a root-closed, asymptomatic permanent tooth, which treatment option would you typically use?

Direct pulp capping

Pulpotomy

Pulpectomy

If you are exposing the pulp during caries excavation in a root-closed, asymptomatic permanent tooth, what material do you use as the first choice for direct pulp capping?

This item is only displayed if the option "Direct pulp capping" is selected in the question "If you expose the pulp during caries excision in a root-closed, asymptomatic permanent tooth, which treatment option would you typically use?"

Calcium hydroxide, e.g. Dycal, Life

Resin modified calcium hydroxide, e.g.: Prisma VLC Dycal, Ultra-Blend Plus, Calcimol LC

Cavity lining, eg IRM and GIC

Cavity lining (light curing), eg Vitrebond, RMGIC

MTA, ex: ProRoot MTA

Calcium silicate-based cements, e.g.: MTA-Angelus, Biodentin

Machine Translated by Google

Resin modified calcium silicate, e.g. Theracal LC

None, bonds directly to the dentin

Other

What other material?

This item is only displayed if the option "Other" is selected in the question "If you expose the pulp during caries excavation in a root-closed, asymptomatic permanent tooth, what material do you use as your first choice for direct pulp capping?"

What percentage of teeth you do direct capping on (with the material choices above) would you estimate will end up with root canal fillings in the long term?

This item is only displayed if the option "Direct pulp capping" is selected in the question "If you expose the pulp during caries excision in a root-closed, asymptomatic permanent tooth, which treatment option would you typically use?"

(Exposure of pulp during caries excavation)

Why have you chosen a chemical-curing material over a light-curing material for direct pulp capping? (you can select multiple options)

This item is only displayed if the option "Calcium hydroxide, e.g. Dycal, Life or MTA, e.g. ProRoot MTA or Calcium silicate-based cements, e.g. MTA-Angelus, Biodentin" is selected in the question "If you expose the pulp during caries excavation in a root-closed, asymptomatic permanent tooth, which material do you use as the first choice for direct pulp capping?"

(Exposure of pulp during caries excavation)

Satisfied with clinical results of chemically cured capping materials Poor clinical

experience with light-cured capping materials Few clinical studies on

light-cured capping materials Light-cured capping materials are

not available at the clinic.

Resin leads to pulp toxicity

Induction of heat during light curing

Other

What other reason?

This item is only displayed if the option "Other" is selected in the question "Why have you chosen a chemical-curing material over a light-curing material for direct pulp capping? (you can select multiple options)"

Why have you chosen a light-curing material over a chemical-curing material for direct pulp capping?

This item is only displayed if the option "Resin modified calcium hydroxide, e.g. Prisma VLC Dycal, Ultra-Blend Plus, Calcimol LC or Cavity lining (light-cured), e.g. Vitrebond, RMGIC or Resin modified calcium silicate, e.g. Theracal LC" is selected in the question "If you expose the pulp during caries excavation in a root-closed, asymptomatic permanent tooth, which material do you use as the first choice for direct pulp capping?"

(Exposure of pulp during caries excavation)

Satisfied with clinical results of light-cured capping materials

The chemically curing capping materials are difficult to use

Clinical studies show better or equal results for light-cured capping materials

Poor clinical experience with the chemically cured capping materials

The light-curing capping materials bond better to permanent filling materials

Other

What other reason?

This item is only displayed if the option "Other" is selected in the question "Why have you chosen a light-curing material over a chemical-curing material for direct pulp capping?"

In the event of mechanical pulp exposure (e.g. trauma, iatrogenic) in a root-closed, asymptomatic permanent tooth, which treatment option would you usually consider?

Machine Translated by Google

use?

- Direct pulp capping
- Pulpotomy
- Pulpectomy

In the event of mechanical exposure of the pulp (e.g. trauma, iatrogenic) in a root-closed, asymptomatic permanent tooth, which treatment option would you usually use, which materials do you use as the first choice for direct pulp capping?

This item is only displayed if the option "Direct pulp capping" is selected in the question "In the event of mechanical exposure of the pulp (e.g. trauma, iatrogenic) in a root-closed, asymptomatic permanent tooth, which treatment option would you usually use?"

- Calcium hydroxide, e.g. Dycal, Life
- Resin modified calcium hydroxide, e.g.: Prisma VLC Dycal, Ultra-Blend Plus, Calcimol LC
- Cavity lining, e.g. IRM, GIC
- Cavity lining (light curing), eg Vitrebond, RMGIC
- MTA, ex: ProRoot MTA
- Calcium silicate-based cements, e.g.: MTA-Angelus, Biodentin
- Resin modified calcium silicate, e.g. Theracal LC
- None, bonds directly to the dentin
- Other

What other material?

This element is only displayed if the option "Other" is selected in the question "In the event of mechanical exposure of the pulp (e.g. trauma, iatrogenic) in a root-closed, asymptomatic permanent tooth, which treatment option would you usually use, which materials do you use as the first choice for direct pulp capping?"

What percentage of teeth you do direct capping on (with the material choices above) would you estimate will end up with root canal fillings in the long term?

This element is only displayed if the option "Calcium hydroxide, e.g. Dycal, Life or Resin modified calcium hydroxide, e.g. Prisma VLC Dycal, Ultra-Blend Plus, Calcimol LC or Cavity lining, e.g. IRM, GIC or Cavity lining (light-cured), e.g. Vitrebond, RMGIC or MTA, e.g. ProRoot MTA or Calcium silicate-based cements, e.g. MTA-Angelus, Biodentin or Resin modified calcium silicate, e.g. Theracal LC or None, bonds directly to the dentin or Other" is selected in the question "In the event of a mechanical exposure of the pulp (e.g. trauma, iatrogenic) in a root-closed, asymptomatic permanent tooth, which treatment option would you usually use, which materials do you use as the first choice for direct pulp capping?"

(Mechanical exposure of the pulp)

Why have you chosen a chemical-curing material over a light-curing material for direct pulp capping?

This element is only displayed if the option "Calcium hydroxide, e.g. Dycal, Life or MTA, e.g. ProRoot MTA or Calcium silicate-based cements, e.g. MTA-Angelus, Biodentin" is selected in the question "In the event of mechanical exposure of the pulp (e.g. trauma, iatrogenic) in a root-closed, asymptomatic permanent tooth, which treatment option would you usually use, which materials do you use as first choice for direct pulp capping?"

(Mechanical exposure of the pulp)

Satisfied with clinical results of chemical-curing capping materials Poor clinical
experience with light-curing capping materials Few clinical studies on
light-curing capping materials Light-curing capping materials are
not available at the clinic.

- Resin leads to pulp toxicity
- Induction of heat during light curing
- Other

What other reason?

Machine Translated by Google

This item is only displayed if the option "Other" is selected in the question "Why have you chosen a chemical-curing material over a light-curing material for direct pulp capping?"

Why have you chosen a light-curing material over a chemical-curing material for direct pulp capping?

This element is only displayed if the option "Resin modified calcium hydroxide, e.g. Prisma VLC Dycal, Ultra-Blend Plus, Calcimol LC or Cavity lining (light-cured), e.g. Vitrebond, RMGIC or Resin modified calcium silicate, e.g. Theracal LC" is selected in the question "In the event of mechanical exposure of the pulp (e.g. trauma, iatrogenic) in a root-closed, asymptomatic permanent tooth, which treatment option would you usually use, which materials do you use as first choice for direct pulp capping?"

(Mechanical exposure of the pulp)

- Satisfied with clinical results of light-cured capping materials
- The chemically curing capping materials are difficult to use
- Clinical studies show better or equal results for light-cured capping materials
- Poor clinical experience with the chemically curing capping materials
- The light-curing capping materials bond better to permanent filling materials
- Other

What other reason?

This item is only displayed if the option "Other" is selected in the question "Why have you chosen a light-curing material over a chemical-curing material for direct pulp capping?"
